# A Multiprofessional and Intersectoral Working Model to Detect and Support Preschool Children With Neurodevelopmental Difficulties (PLUSS Model): Protocol for an Evaluation Study

**DOI:** 10.2196/34969

**Published:** 2022-06-15

**Authors:** Berit M Gustafsson, Laura Korhonen

**Affiliations:** 1 Linköping University Department of Child and Adolescent Psychiatry Department of Biomedical and Clinical Sciences Linkoping Sweden

**Keywords:** early detection, early intervention, preschool children, multiprofessional, neurodevelopmental difficulties, parental support, preschool support, mental health, neurological, behavioural, emotional, paediatrics, pediatrics, parenting, children, neurodevelopmental, developmental

## Abstract

**Background:**

Neurodevelopmental difficulties with various emotional and behavioral symptoms increase the risk of mental health problems later in life. Although we know that early detection and interventions are effective, there is a lack of intersectoral, integrative, and evidence-based working models to provide these services for preschool children and their parents. PLUSS (*Psykisk hälsa Lärande Utveckling Samverkan kring Små barn*; English translation: mental health, learning, development, collaboration around preschool children) is a collaborative “one way in” model involving parents, health care providers, preschools, social services, and researchers. PLUSS provides coordinated services to screen, evaluate, and support toddlers with neurodevelopmental problems. It also offers parental interventions and education for preschool teachers.

**Objective:**

The model will be studied in a research project that aims to investigate (1) using a quasi-experimental study on longitudinal trajectories of neurodevelopmental difficulties and ability to function among participating preschoolers, (2) user satisfaction, and (3) implementation of the model and its effectiveness. The long-term goal is to provide evidence-based, coordinated services to reduce problems related to neurodevelopmental difficulties among preschool children and promote well-being and functioning in everyday life.

**Methods:**

The population of interest is children aged 1.5-5 years, whom the child health care nurse refers for further assessment due to suspected neurodevelopmental problems. Data are collected using questionnaires and semistructured interviews. Measures include sociodemographic data, longitudinal data on neurodevelopmental problems, parental well-being and satisfaction, the effectiveness of parental and preschool teacher training and implementation of the model, and fostered multisectoral collaborations. Data will be analyzed with qualitative and quantitative methods.

**Results:**

The PLUSS model has been approved by the National Ethics Review Board (2019–04839). This study was supported by FUTURUM grants 910161 and 910441. Data collection started in April 2019, with the data collection period planned to end in May 2024.

**Conclusions:**

PLUSS is an integrative working model with multiprofessional competence and intersectoral collaboration capacity to help preschool children with neurodevelopmental problems and their parents. It will be studied using quasi-experimental cross-sectional and longitudinal study designs. Data will be collected from parents, health care providers, and preschool teachers, and will be analyzed using quantitative and qualitative methods. The study will run in one Swedish county, and generalizability needs to be studied separately. Loss of follow-up could impact the longitudinal analysis.

**Trial Registration:**

ClinicalTrials.gov NCT04815889; https://clinicaltrials.gov/ct2/show/NCT04815889

**International Registered Report Identifier (IRRID):**

DERR1-10.2196/34969

## Introduction

### Background

Neurodevelopmental problems among preschool children are common, with an estimated 7%-10% prevalence [1]. Early-onset externalizing or internalizing problems predict the later development of mental health problems, corresponding to or overlapping with the initial symptoms [1,2]. The earlier behavioral problems occur in a child’s life, the greater the risk [3,4].

Early identification of children with neurodevelopmental problems is crucial for providing adequate support [1,5]. Despite this knowledge, several Swedish reports have highlighted a dire need for research into the mental health of children of preschool age [6-8]. For example, data on the current prevalence of significant neurodevelopmental problems among Swedish children aged 0-5 years are missing. Inherent to this is how many of these children have been offered and are receiving any kind of intervention. In addition, studies on longitudinal trajectories of problems and provided services are scarce.

In Sweden, child health care is responsible for the early detection and follow-up of developmental problems among preschool children [9]. Swedish child health care has a well-established screening program that reaches 95% of all preschool children [10]. In addition, preschools serve as an environment where children’s problems can be identified, promoting good mental health [11]. Collaboration between child health care and preschools provides a significant opportunity to identify children and families needing support and treatment [12,13]. Standardized assessment methods, such as questionnaires, scales, and observations, with proven reliability are one way to further facilitate the early detection of emotional and behavioral problems related to neurodevelopment [14].

Despite the established health monitoring system, there is no homogeneous system of mental health services for children below school age in Sweden. Regional differences exist, and both public and private service providers are involved. Some children are referred to Child and Adolescent Psychiatry clinics, while others are assessed and followed up on by child habilitation or municipal counseling units [15]. There are also well-known shortcomings in collaborations between health actors and other multisectoral partners such as social welfare services [4]. Coordination of efforts is difficult to establish, and queues of several years for assessment and treatment are frequent.

Efforts to strengthen overall mental health in preschool children appear to have a positive effect later in life [1,16]. A preschool with adequate resources is suitable for health promotion and prevention, with learning opportunities that increase children’s social, cognitive, and adaptive skills [17]. In addition, several studies have shown that group-based parent support programs can improve emotional and behavioral problems among preschool children. However, the long-term efficacy of these programs is uncertain, as are their primary prevention effects [18].

### Theoretical Background of the Study

Theoretically, this project is based on Bronfenbrenner’s socioecological model. According to this model, a child’s development is, apart from genes, influenced by various microsystems (family, preschool teachers, peers, etc), the ecosystem’s support of the family, and preschool structure, as well as by macrosystem-level laws, culture, and policies [19]. Here, concepts such as “person-process-context-time” are highlighted in the proximal process where the person (preschool child) in their approach (play) in his context (preschool/family) develops and learns, for example, interaction over time [20]. Early support to the child, parents, and preschool teachers is expected to promote positive development and increased everyday functioning over time.

A child’s behavior undergoes age-related developmental changes, including progress in motor skills, language, self-esteem, and how to handle emotional regulation. One fundamental skill is self-regulation. It is a multilevel construct that describes the ability of an individual to optimally manage physiological arousal, emotions, attention, behavior, and cognition. Self-regulation helps the child acquire the behavioral, emotional, and cognitive self-control essential for competent functioning and autonomy, both in childhood and life [21]. Acquired developmental skills also support a child’s functioning in everyday life, independent of age. There is an interplay between genes and environmental factors throughout life, including parental support, attachment to caregivers, and the child’s emotional experiences [21]. Furthermore, interactions among preschool children are essential for developing cognitive regulation and coping skills and play a part in equipping children to handle demanding experiences in life [22,23]. Theories of risk and protective factors have determined that it is essential to increase dynamic/impactful health factors as early as possible and reduce the number of risk factors in the child’s context [2].

The overall aim of this study is to study the PLUSS (*Psykisk hälsa Lärande Utveckling Samverkan kring Små barn*; English translation: mental health, learning, development, collaboration around preschool children) model that provides coordinated services to screen, evaluate, and support children (aged 1.5-5 years) with neurodevelopmental problems. The project has the following specific aims:

1. To study neurodevelopmental issues and the ability to function among preschool children longitudinally.
2. To study parental well-being and satisfaction with provided and used services.
3. To study the implementation of the model and its effectiveness, including parental and preschool teacher training and multisectoral collaborations.

We hypothesize that a coordinated working model with multiprofessional and intersectoral collaborations will promote early detection and support of preschool children with neurodevelopmental problems. We expect that this will also positively impact mental health and well-being in the long run. In addition, we hope that this working model enhances user satisfaction and the effectiveness of processes to provide services.

## Methods

### Setting and Data Collection

**Figure 1:**
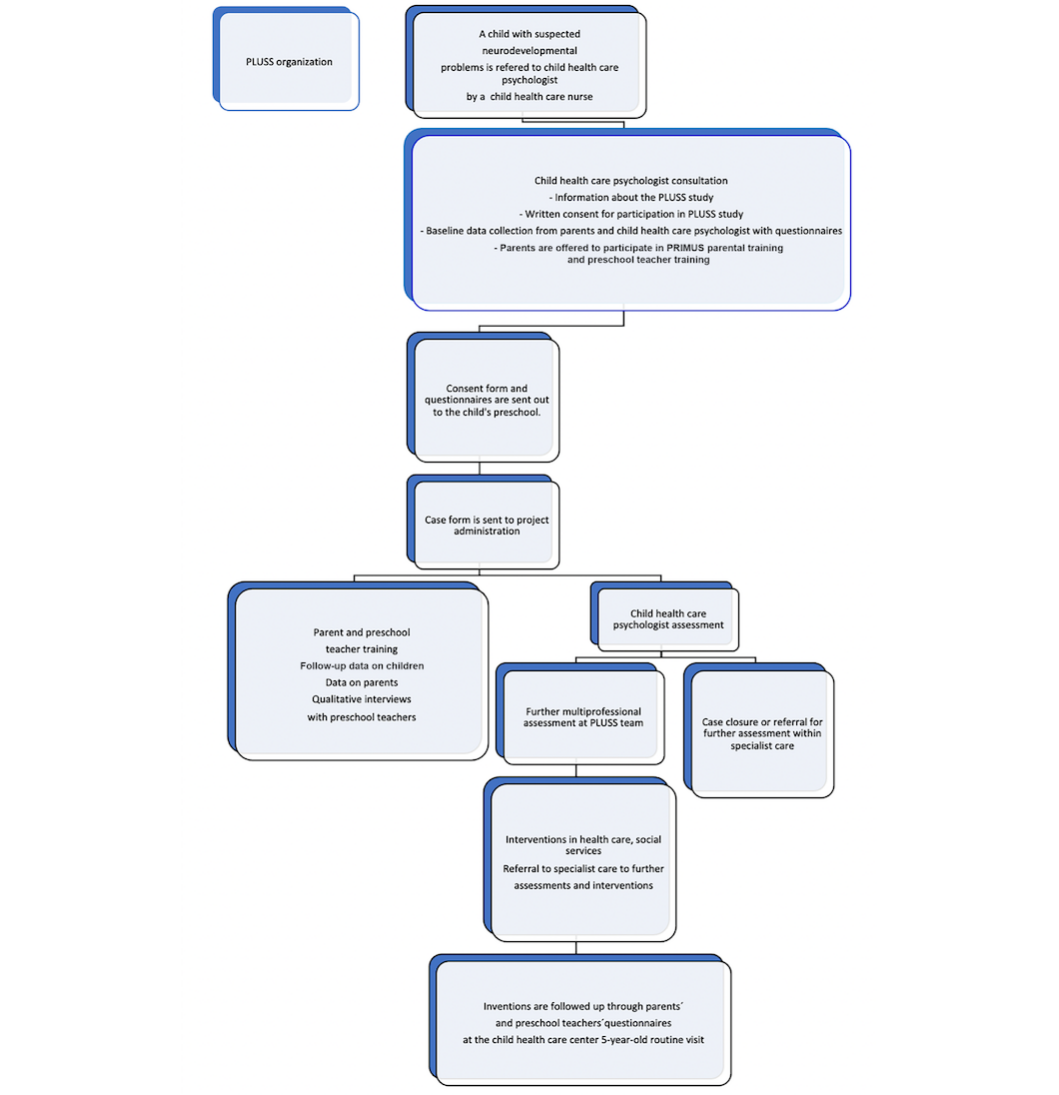
The PLUSS procedure. PLUSS: *Psykisk hälsa Lärande Utveckling Samverkan kring Små barn* (English translation: mental health, learning, development, collaboration around preschool children).

The study is based in Jönköping County, in the south of Sweden, and runs within the PLUSS project. The PLUSS model is built upon existing processes for patient flow, from early detection to assessment and interventions. Figure 1 shows the PLUSS flow. Parents of children referred to child health care psychologists due to neurodevelopmental problems are informed about the study by child health care nurses. Parents sign a consent form for participation and fill out questionnaires before the child’s health care psychologist consultation. Subsequently, parents are offered the possibility to participate in a parental training program (PRIMUS). Data are also collected from preschools and preschool teachers who are offered a separate training program. Following the child health care psychologist assessment, and initial parental and preschool teacher training, the child health care psychologist consults the multiprofessional PLUSS to plan and coordinate further evaluations and interventions.

### Study Design and Populations

Inclusion criteria include the following: a child referred to a child health care psychologist, aged 1.5-5 years, with ESSENCE (Early Symptomatic Syndromes Eliciting Neurodevelopmental Clinical Examinations) problems such as developmental delay, interaction, contact difficulties, language and communication difficulties, difficulties in everyday function, concentration/hyperactivity, self-regulation, acting/boundaries, and anxiety [1]. Exclusion criteria include the following: the family only needs parental support and/or there is a risk that participation in PLUSS would delay assessments or referrals to specialized services.

Detailed information about the study is given below. Research within PLUSS focuses on children, parents, and preschool teachers, and professionals working with the PLUSS are included in some substudies. Both cross-sectional and longitudinal quasi-experimental study designs are used. One substudy uses focus group interviews.

### Sample Size

The aim is to collect data on 700 children. This sample size allows proposed analyses of primary outcomes related to the Strengths and Difficulties (SDQ) instrument as well as subgroup analysis and person-based analysis (cluster analysis and path analysis) with statistical strength of 0.80 and α<.05. Power estimation is based on previous studies by Rothenberger et al [24]. The estimated number of children in the PLUSS pilot project during the first year is 160 children, and for Jönköping County, approximately 860 children per year. Studies on parental training and the user study have estimated sample sizes of n=160 and n=100, respectively. For qualitative studies, a sample size of n=20 is used [25]. Finally, the implementation study will collect data from n=80 professionals and n=100 parents involved in the PLUSS.

The control group (n=160) will be recruited from the parts of the county that have not yet been included in the PLUSS. This means that children/families belonging to the control group receive treatment as usual.

### Instruments

Child health care nurses, psychologists, and preschool teachers serve as informants. Behavioral problems are measured with the SDQ (25 items and a supplement with 8 items). SDQ is validated, and proposed cutoffs are available for Swedish conditions [14,26,27]. The Children’s Engagement Questionnaire (CEQ; 29 items) measures preschool children’s targeted engagement and social interaction in everyday life [28,29]. The Joint Attention Observation tool (JA-OBS; 5 items) screens for the autism spectrum [30]. Psychosocial problems in the family are measured with LAPS (*lapsen psykososiaalisen terveyden arviointimenetelmä*; English translation: child mental health assessment form) [31]. A self-constructed questionnaire collects sociodemographic and background questions, including questions on mother tongue, possible diagnosis, education, professional activities, and parental ability. The health care process, for example, the number of investigations, waiting time for assessment, visits, and satisfaction, is also compiled with a separate questionnaire. Collaboration between professionals in different organizations is assessed using the “Spider” measurement, which has 10 questions [32].

### Data Analysis

Table 1 summarizes the primary outcomes, confounders, and analysis that will be conducted. Quantitative multivariate analyses will be done in SPSS (version 28; IBM Corp), and longitudinal data will be analyzed using person- and group-centered analytic methods, cluster analysis, and path analysis. A comparison study with the control group and children referred to habilitation centers will occur. The focus group and semistructured interviews will be analyzed qualitatively with content analysis.

**Table 1:** Summary of substudies included in the PLUSS.^a^

Study (design and time frame)	Study population	Informants (instruments)	Main outcomes
User study (qualitative focus group interview study, conducted 2019-2020)	Parents of a 1.5- to 5-year-old child with neurodevelopmental problems (n=13)	Parents (data collected with semistructured focus group interviews)	Satisfaction with health care system
Pilot study on children with neurodevelopmental problems (quasi-experimental quantitative study, conducted 2020-2021)	1.5- to 5-year-old children with neurodevelopmental problems (n=80)	Parents, child health care psychologists, preschool teachers (data collected with SDQ, LAPS, CEQ, JA-OBS, background questions, medical records)	Neurodevelopmental and mental health–related problems: development delay, interaction, contact difficulties, language and communication difficulties, motor difficulties, concentration/hyperactive, self-regulation, acting/boundaries, anxiety Difficulties in everyday function Psychosocial stress factors Socioeconomic status, family constellation, mother tongue, possible diagnosis, education, professional activities The health care process indicators (eg, number of assessments, visits, interventions)
Full-scale study on children with neurodevelopmental problems (quasi-experimental quantitative study with longitudinal follow-up, conducted 2022-2024)	1.5- to 5-year-old children with neurodevelopmental problems (n=160), compared to treatment as usual (n=160)	Parents, child health care psychologists, preschool teachers (data collected with SDQ, LAPS, CEQ, JA-OBS, background questions)	As above
PRIMUS parental training program (cross-sectional quantitative study, conducted 2022-2024)	Parents to a 1.5-5-year-old child with neurodevelopmental problems (n=160)	Parents (data collected with PRIMUS evaluation questionnaire, SDQ, CEQ, LAPS)	Self-rated parental ability Neurodevelopmental and mental health–related problems: development delay, interaction, contact difficulties, language and communication difficulties, motor difficulties, concentration/hyperactive, self-regulation, acting/boundaries, anxiety Difficulties in everyday function Psychosocial stress factors

^a^CEQ: Children’s Engagement Questionnaire; JA-OBS: Joint Attention Observation tool; LAPS:*lapsen psykososiaalisen terveyden arviointimenetelmä* (child mental health assessment form); PLUSS: *Psykisk hälsa Lärande Utveckling Samverkan kring Små barn* (mental health, learning, development, collaboration around preschool children); SDQ: Strengths and Difficulties Questionnaire.

### Ethics Approval

Ethics approval has been granted by the National Ethics Board (2019-04839). Informed consent is obtained from all actors: parents, managers, preschool educators, child health care psychologists, and child health care nurses. All data are registered with a participant number and encoded directly at the time of collection, considering privacy protection. The code template for translation between participant number and the test subject can be found in a logbook inaccessible to unauthorized persons. The results are reported only at the group level, where no personal data will be recognizable. All data processing follows the Swedish data law. The parents have been informed that their children will receive the standard care even if they do not participate in the study. Upon parents’ informed consent, the preschool manager and preschool teacher may consent to participate in the research, and the preschool teacher answers questionnaires.

## Results

All research included in the PLUSS model has been approved by the National Ethics Review Board (2019–04839). Informed consent will be obtained from all study participants and legal guardians if the participant is younger than 15 years. Results will be available to caregivers, professionals working with preschool children, researchers, and funders.

This study was supported by FUTURUM grants 910161 and 910441. The funders had no role in designing the study, writing the report, or deciding to submit the paper for publication.

The study has been registered at ClinicalTrials.gov (NCT04815889). The data collection for the pilot study started in April 2019, with the prior data collection period finished by April 2021. Data collection for the full-scale investigation began during May 2021 and is planned to be completed in May 2024.

## Discussion

### Principal Findings

Expected results from this study include estimates of the prevalence of neurodevelopmental problems in preschoolers, their impact on functional ability, and parental well-being. In addition, the effectiveness of the PLUSS working model is elucidated. Qualitative studies are expected to give us information about parents’ and preschool teachers’ experiences related to children with neurodevelopmental problems and how the system can support these children.

A research project conducted within ongoing clinical work provides an excellent opportunity to improve health care but is also affected by everyday obstacles. Not all activities and departments are accustomed to participating in clinical research work, and additional assignments for staff might cause skepticism to arise. The longitudinal study design will allow an analysis of changes over time in the same study participant, providing more substantial evidence for causality than could be obtained from a cross-sectional design. However, loss to follow-up may occur. Combining quantitative and qualitative approaches provides an excellent opportunity to understand the actual change in a child’s behavior with satisfaction and well-being. Another strength is that collaboration and organizational measures are studied in PLUSS.

The project is based at Jönköping County’s health care, collaborating with preschools and social services and researchers from Linköping University. Children and parents are not directly involved in the study design, recruitment, or conduct of the research. Parents are, however, engaged in focus groups that give, for example, input on how the parent educating group should be designed and what it should contain. The focus groups will involve parents of children with neurodevelopmental problems and obtain support from child health care and specialist health care. This study will be made available to the participants, the funders, professionals, researchers, and policy makers.

An important aim for future research in the PLUSS model is to follow the included children long term regarding symptom development, set diagnoses, and evaluate given interventions. Parental stress and perceived competence of parents of children with neurodevelopmental difficulties will also be considered in future research.

To improve children’s mental health in Sweden, 5 suggested, evidence-based interventions were recently published, one of them being early detection and early interventions for young people at risk for future mental health problems [33]. Current knowledge of early detection and early interventions for children suggests they have been beneficial long term and economically justifiable [5,34]. The PLUSS model has been constructed to better meet the demands of health care today with early interventions for parents and children exhibiting neurodevelopmental difficulties.

Information about the ongoing study and obtained results will be communicated via the PLUSS project’s steering group and working group within and outside the Jönköping County Region. The scientific results will be disseminated to the caregivers, professionals working with preschool children, researchers, and funders. Community policy makers and stakeholders will be targeted separately using Jönköping County’s existing information channels and conferences.

### Conclusions

PLUSS is an integrative working model with multiprofessional competence and intersectoral collaboration capacity to help preschool children with neurodevelopmental problems and their parents. The study has a quasi-experimental cross-sectional and longitudinal design. Data are collected from parents, health care providers, and preschool teachers and analyzed using quantitative and qualitative methods. The study is run in one Swedish county, and generalizability needs to be studied separately.

## Abbreviations

CEQ: Children’s Engagement Questionnaire
ESSENCE: Early Symptomatic Syndromes Eliciting Neurodevelopmental Clinical Examinations
JA-OBS: Joint Attention Observation tool
LAPS: *lapsen psykososiaalisen terveyden arviointimenetelmä* (child mental health assessment form)
PLUSS: *Psykisk hälsa Lärande Utveckling Samverkan kring Små barn* (mental health, learning, development, collaboration around preschool children)
SDQ: Strengths and Difficulties Questionnaire

## Data Availability

Data collection is ongoing. The results from this project will be published in open access journals. The data sets generated during this study will be available from the corresponding author on reasonable request.
